# Advanced Implant-Prosthetic Rehabilitation: How to Obtain a Correct Restoration of Both Functions and Aesthetics in Patients with Complex Combined Dental and Maxillofacial Trauma: A Case Report and Topical Review of the Literature

**DOI:** 10.1155/2017/7146126

**Published:** 2017-03-14

**Authors:** M. M. Figliuzzi, A. Giudice, L. Fortunato

**Affiliations:** Department of Periodontics and Oral Sciences, “Magna Graecia” University, 88100 Catanzaro, Italy

## Abstract

*Aim*. This study aims to explain the main steps that characterize the implant-prosthetic rehabilitation in complex combined dental and maxillofacial trauma.* Material and Methods*. A 20-year-old patient reported an extensive facial trauma which also involved the alveolar process of the maxillary bone. The patient reported a maxillofacial fracture and the loss of teeth 1.3, 1.2, 1.1, and 2.1. A “Le Fort” type 2 fracture was also reported, with the malar bone involvement. After reduction and containment of bone fractures, through appropriate mounting plates, appropriate functional and aesthetic rehabilitation of the patient were replaced thanks to a temporary removable prosthesis. After 6 months, the patient performed numerous clinical investigations, aimed at a proper planning of implant-prosthetic rehabilitation of the upper dental arch.* Conclusion*. With the planning of the case, as well as respecting the surrounding biological structures, the surgery of implants can be carried out with the most appropriate procedure. Lastly, new dental implants with highly bioactive surfaces have been developed, providing an excellent and rapid bone integration.

## 1. Introduction

One of the most common consequences of road accidents is the maxillofacial trauma with involvement of one or both dental arches. The surgical approach of maxillofacial trauma must follow a surgical procedure, requiring systemic evaluations such as the hemodynamic evaluation, the correct assessment of the wound, the presence of foreign bodies, the lesions involving nerves or vessels or glandular ducts, and other evaluations which must be carefully evaluated with preoperative instrumental examinations [1].

Surgical treatment of trauma that also involves one or more dental elements is a complex activity, often performed in several stages, depending on the type of the injury and the age of the patient.

The most commonly involved teeth in such trauma are the upper central incisors, due to their length, their position, and the scarce protection by the lips. These are subjected to fracture approximately ten times more than the lower incisors. In these cases, surgeons and dentists must restore both the masticatory function and the anatomy of the mouth [2, 3].

Traumatic avulsions are treated with a mobile or fixed prosthetic rehabilitation. The permanent fixed prosthesis is preferable, as it can ensure a greater functionality, the right comfort, and an extremely positive aesthetic result to the patient [4].

Indeed, an implant-prosthetic rehabilitation following a dental and maxillofacial trauma can cause numerous complications; therefore, the planning of the aesthetic and functional rehabilitation should be prepared meticulously.

It is necessary to ensure an emergency profile of the required dental implants. To ensure a correct prosthetic rehabilitation from both the functional and aesthetic point of view, the surgeon must carefully evaluate the volume of residual bone tissue, consequently planning the implants placement, preferably by using a prosthesis-guided approach [5].

This study aims to explain the main steps that characterize the implant-prosthetic rehabilitation in such cases of dental and maxillofacial trauma. Here, the case of a 20-year-old boy was reported: the patient reported a severe and complex facial trauma, with the fracture of several frontal teeth, with functional and aesthetical issues 1.3, 1.2, 1.1, and 2.1.

## 2. Material and Methods

**Figure 1:**
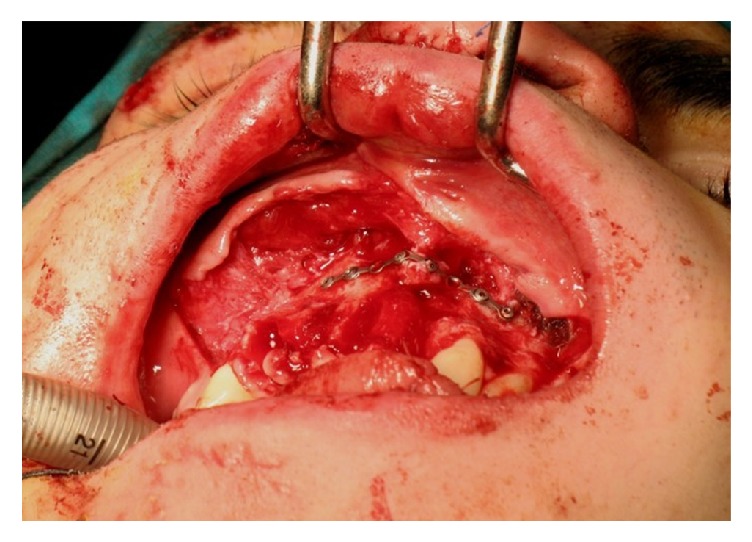
Clinical vision of the patient with a maxillofacial fracture and the loss of teeth.

Following a car accident, the twenty-year-old patient reported extensive facial trauma which also involved the alveolar process of the maxillary bone. The patient reported a maxillofacial fracture and the loss of teeth 1.3, 1.2, 1.1, and 2.1. A “Le Fort” type 2 fracture was also reported, with the malar bone involvement (Figure 1).

**Figure 2:**
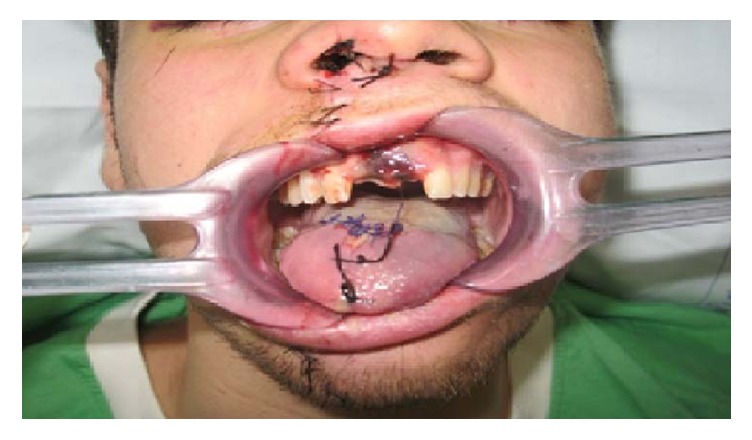
Reduction and containment of bone fractures through appropriate mounting plates.

The patient was hospitalized in Maxillofacial Surgery of the University “Magna Graecia” in Catanzaro. After being admitted to hospital, the patient underwent maxillofacial surgery: obtaining the reduction and containment of bone fractures through appropriate mounting plates (Figure 2).

To ensure an appropriate functional and aesthetic rehabilitation of the patient, the missing dental elements were replaced thanks to a temporary removable prosthesis.

**Figure 3:**
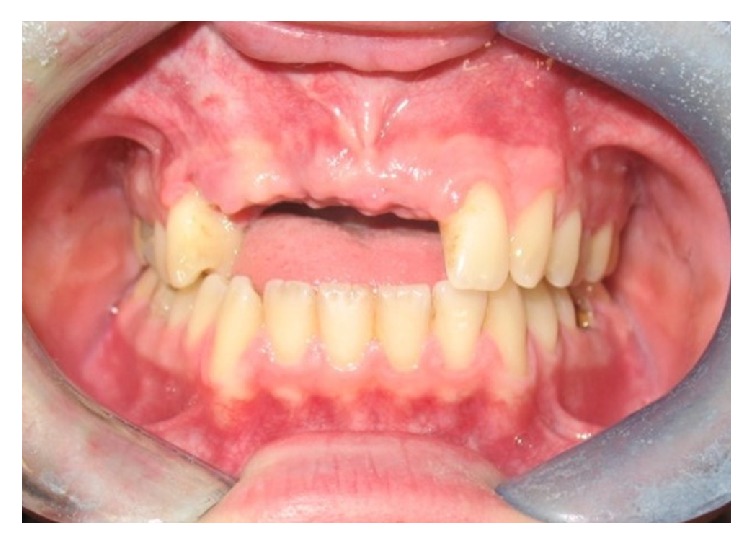
Clinical vision after 6 months.

**Figure 4:**
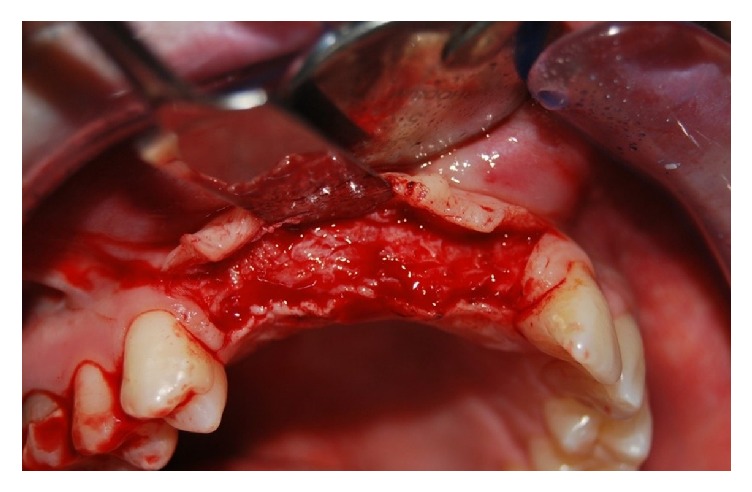
Incision with partial-thickness flap after 6 months.

After six months, the patient has performed numerous clinical investigations, aimed at a proper planning of implant-prosthetic rehabilitation of the upper dental arch (Figures 3 and 4).

The Cone-beam CT is performed with the aim to evaluate the residual bone thickness: this allows us to plan an optimal inclination of the implant fixtures, in relation to the dental prostheses that will be placed on the implants.

The removable prosthesis is used as a surgical guide: this technique is commonly described as a “prosthesis-guided implant surgery.”

The surgical technique was performed with the execution of a partial-thickness flap; this type of mucogingival flap has endured the in situ maintenance of the periosteum: guaranteeing an appropriate blood supply to the surgical site during all the stages of implant surgery.

The operating protocol is schematized as described below:

  The first stage is a local anesthetic. With the anesthetized tissue, the surgeon performs a partial-thickness flap with the blade type Beaver 64, the insertion technique of the implants.
  The testing of implant parallelism is essential for a correct insertion of the prosthesis on implants, at a later date.
  The final suture of the flap should be performed with the surgical staples firmly anchored to the periosteum.

## 3. Case Report

A case is reported with the intent to describe the technique performed in this research.

**Figure 5:**
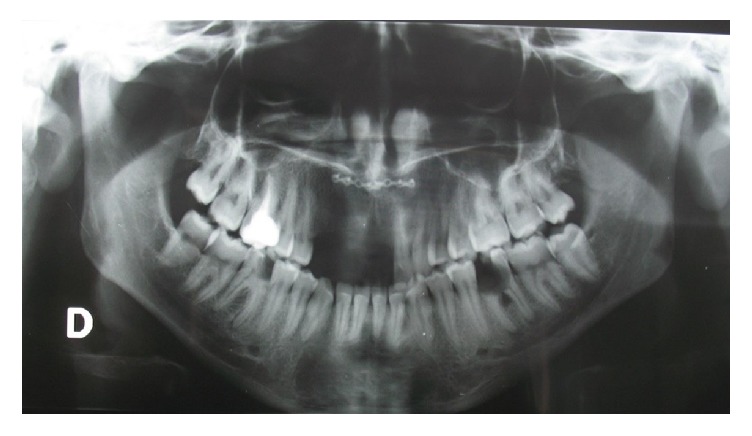
Orthopanoramic view after 6 months.

The surgical procedure is performed with an initial local anesthetic. The mucosa is cut, with a 64 Beaver blade, onto the gingival region that covers the alveolar bone crest but without reaching the periosteum, to have a residual thickness anchored to the periosteal vascular network. The crestal incision is delimited by two accessory incisions to ensure the correct mobility of the flap, both mesial and distal. The two additional incisions are divergent, so as to ensure an adequate blood supply, promoting optimal healing of the tissues (Figure 5).

**Figure 6:**
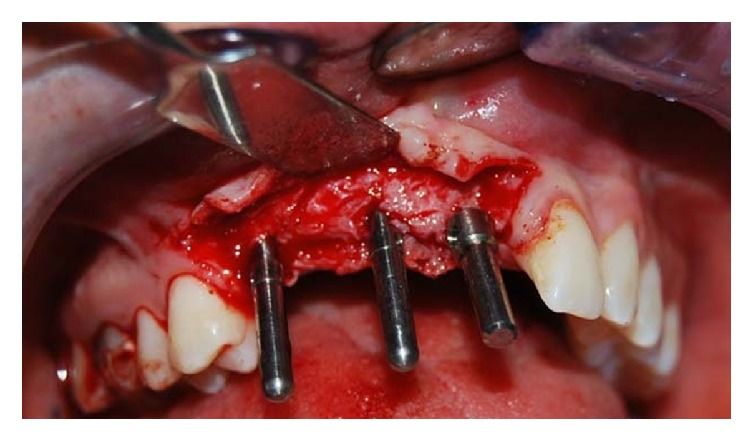
Clinical vision of the parallelism pins.

Following the inclinations outlined by the provisional prosthesis, the bone site for implant placement has been prepared (Figure 6).

**Figure 7:**
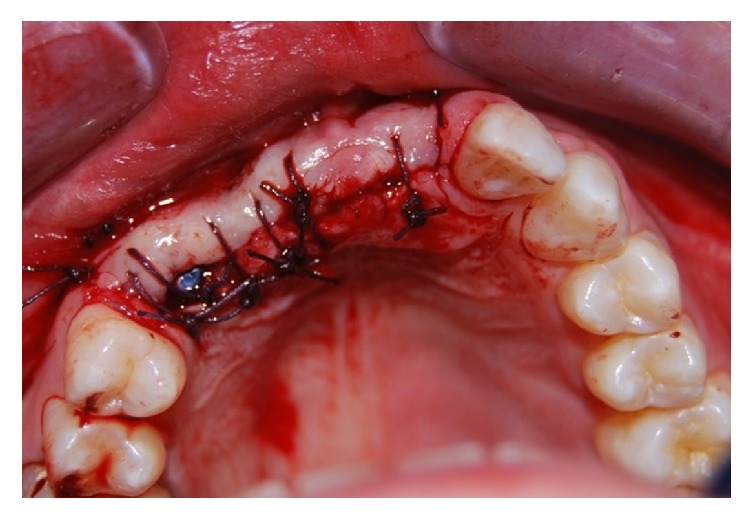
Implants insertion.

**Figure 8:**
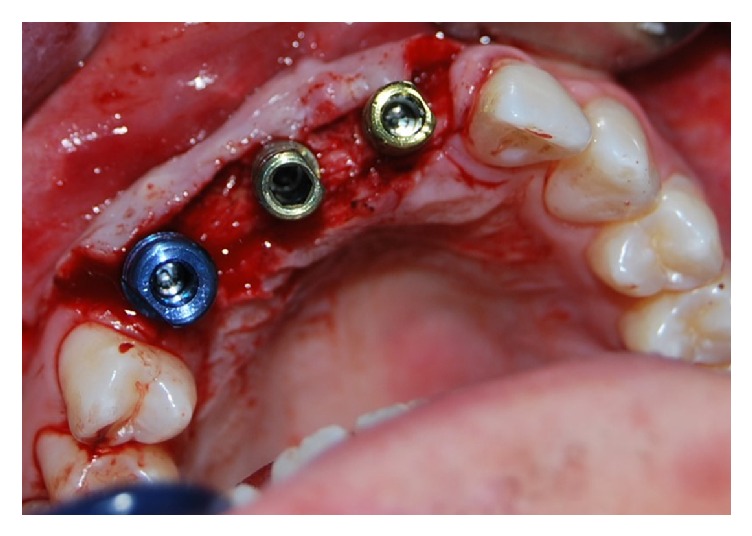
The flap was properly closed with single stitches linking the periosteum.

The implants were inserted in positions 1.3, 1.1, and 2.1: in the area of the elements 2.1 and 1.1, two implants 4 × 11,5 mm were inserted. Instead, in the 1.3 position, a 4,5 × 11,5 mm implant was inserted. In this case report products systems have been used with laser-sintering technology, type “Tixos” (Leader Italy, Cinisello Balsamo, Milan, Italy) (Figure 7). The flap was properly closed with single stitches linking the periosteum (Figure 8).

**Figure 9:**
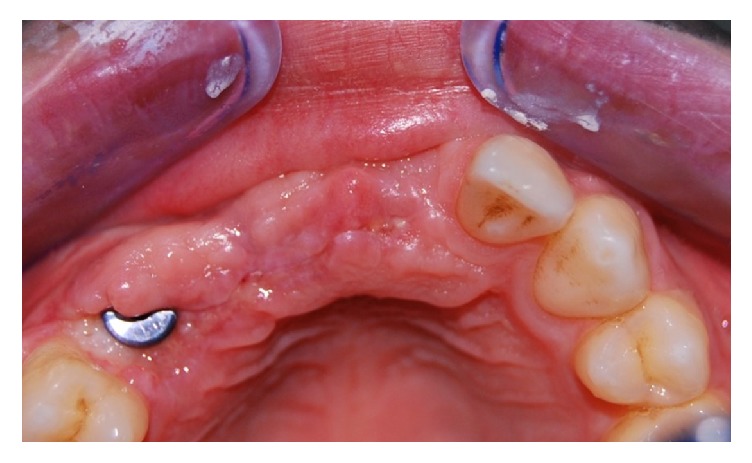
Tissue healing after 15 days.

**Figure 10:**
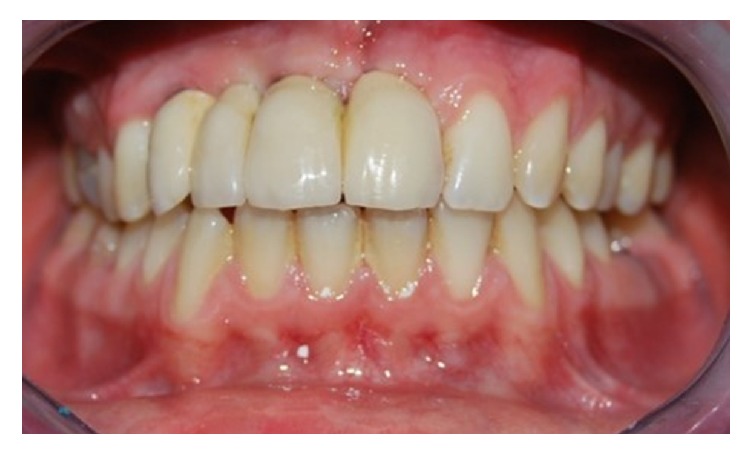
Profiles of the prosthetic abutments.

Following the implant surgery, the patient was provided with a provisional removable prosthesis. After a week, the stitches were removed. Clinical control after 15 days shows an optimal tissue healing (Figure 9). Lastly, three months after implant placement, the final prosthesis was applied on the implants (Figure 10).

**Figure 11:**
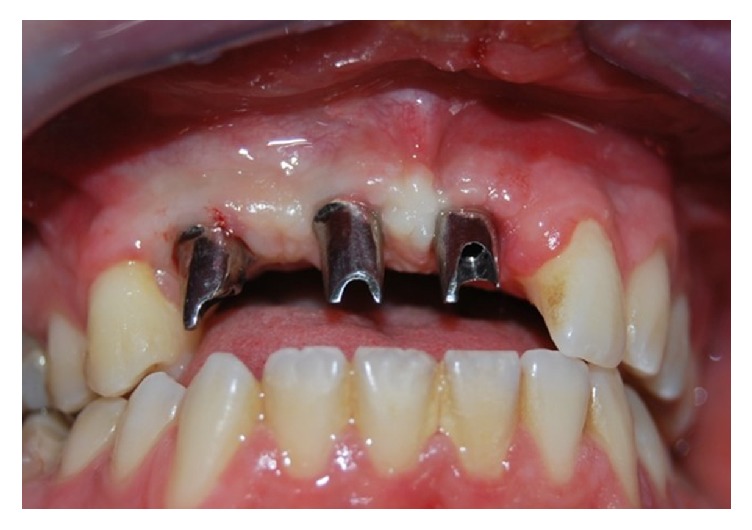
Clinical control after 6 months.

**Figure 12:**
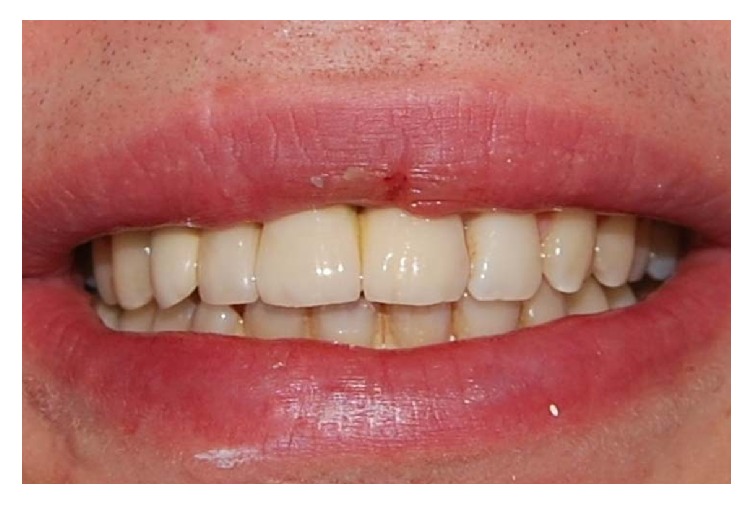
Clinical control after 6 months.

Following the application of the permanent prosthesis, clinical and radiological checks were performed regularly at three-month periods (Figures 11 and 12).

**Figure 13:**
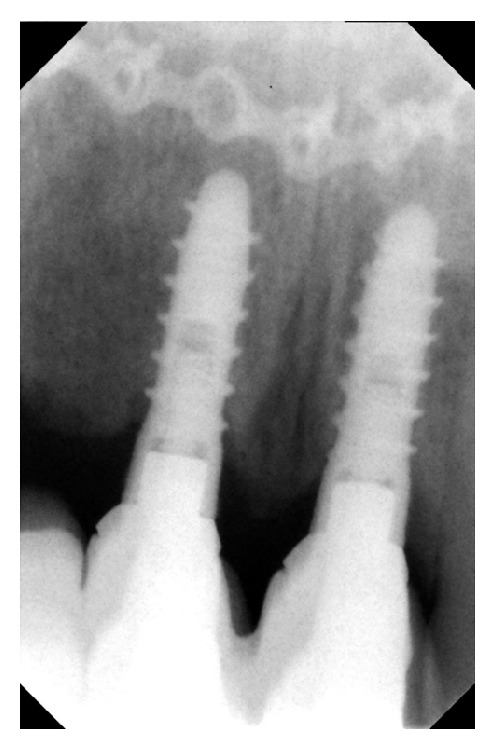
Radiological control after five years.

**Figure 14:**
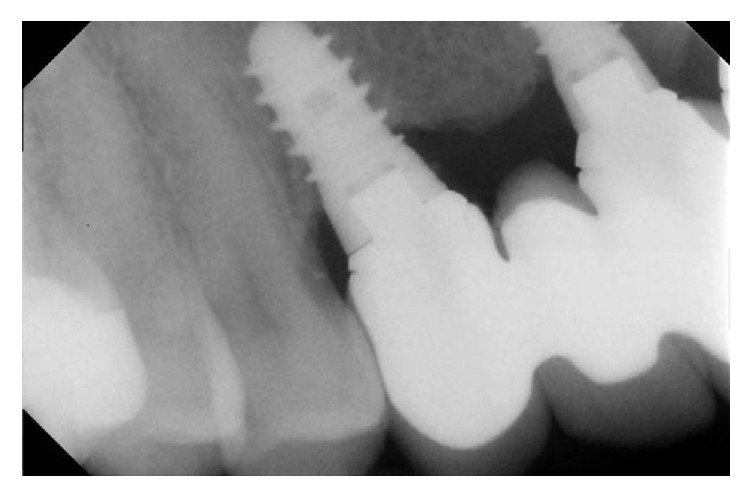
Radiological control after five years.

**Figure 15:**
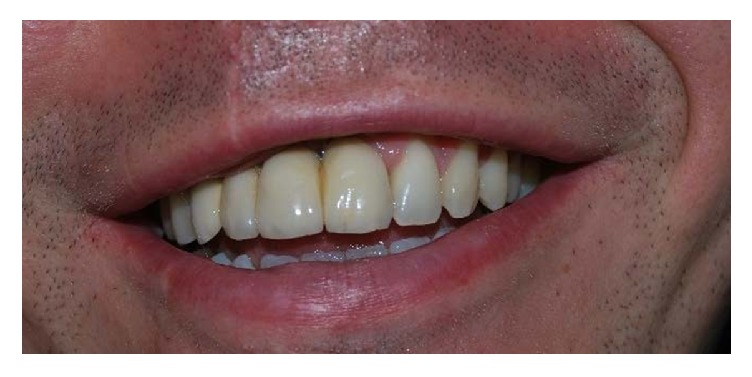
Clinical control after five years.

**Figure 16:**
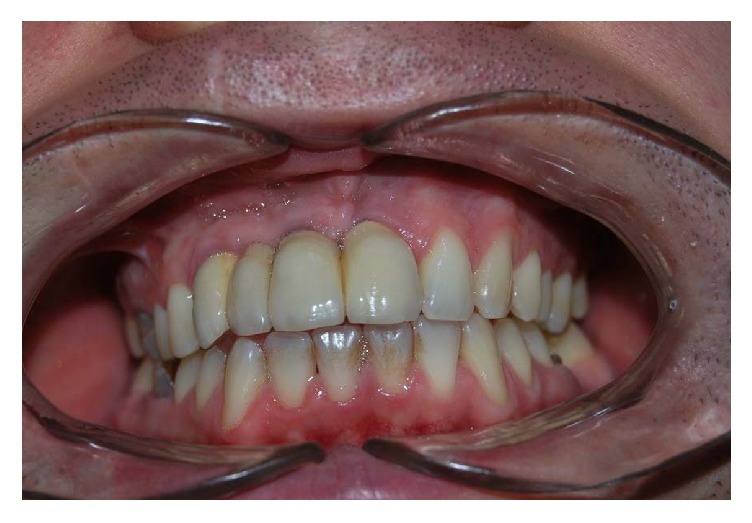
Clinical control after five years.

Following the first year, checks were carried out every 6 months, for the following five years (Figures 13, 14, 15, and 16).

## 4. Discussion

The maxillofacial traumas are the causes of many aesthetic and functional anomalies [6]. In addition, the teeth and periodontal structures are often seen in facial trauma [7, 8]. In such cases, the rehabilitation must be both dental and maxillofacial, through the implant surgery that can restore the correct morphologies and functions of the maxillofacial area.

After a maxillofacial trauma involving teeth, an exceptional aesthetic rehabilitation must be carried out, respecting set parameters:

- the biological and anatomical features relative to the bone tissue to be treated with surgery;
- use of a minimally invasive surgical technique;
- plan for optimal management of peri-implant soft tissue;
- evaluation of the shape and surface geometry of the type of dental implant required;
- ensuring proper placement of the implant in the bone crest.

A determining factor for a good bone integration of implants is to have a quantity of bone that measures at least 2 mm around the implant [9, 10].

In addition, it is necessary to ensure a good “emergence profile,” to ensure a correct aesthetic and functional management of the following dental prosthesis [11].

Therefore, it is very important to plan the implant-prosthetic rehabilitation: such planning can be done only if the bone thickness where the implants will be inserted is known. These sizes can be established through commonly used technologies, such as Cone-beam CT. To schedule the permanent prosthesis model, using the indications the provisional prosthesis gives us, it is an optimal surgical guide for implant placement.

In those cases where the bone thickness is insufficient to ensure proper implant stability, the techniques of guided bone regeneration (GBR) can be used [12].

Many authors [13] have proposed a GBR through the use of bone grafts directly from intraoral sites (e.g., the ascending branch of the mandible); on the other hand, other authors have proposed many bone harvesting sites, external to the oral cavity (e.g., the iliac crest). GBR with bone grafts are very invasive for the patient; moreover, they have different critical issues, as the need to perform two surgical procedures, and there will be a double morbidity.

In light of the problems that some authors have reported in using the techniques of bone grafts, other authors have proposed different methods to increase the thickness of the bone crest: the subsequent techniques were performed by separating the buccal and lingual cortical bone (“split-crest” technique). The technique of “split-crest” induced a new bone formation, thus facilitating the subsequent insertion of dental implants [14].

In the scientific literature, some “hybrid” methods can be found: they are performed through the use of “split-crest” technique, associated with the addition of synthetic biomaterials [15].

Of course, in all surgical techniques it is essential to respect the biological principles; in fact, the human body already has all the reparative and regenerative abilities, and these skills are able to reduce the physiological processes of bone resorption, as a result of bone surgery. From this point of view, a partial-thickness flap is used, and there will be greater chance of a better healing of tissues and a reduced bone resorption [16].

In recent years, scientific research paid great attention to the implant surfaces, with the aim of creating the ideal surface, capable of ensuring the optimal osseointegration of the implants.

Recently, a new technology has been described: this technology is called Direct Laser Metal Forming (DLMF) offering many advantages, allowing you to get dental implants with a morphology, size, and structure of the surface, configurable through a 3D software. After having designed the model of our implant, the software will guide the sintering of titanium powder with laser technology, so to obtain the desired implant.

The microstructure of the implant surface made with DLMF technology can have a 3D morphology, with numerous microcavities interconnected between them, which tend to replicate the bone structure, thus, making such surface bioactive. The microcavity typically is between 2 and 200 microns, and the depth of the cavities can be up to 200 microns in the implant structure: these microtunnel and microcavities will be then invaded by bone cells from the surrounding environment. A further feature of the implants created with the DLMF technology is the isoelasticity of the surface: Young's modulus values are very close to those of the natural bone [17]. Many studies on DLMF technology applied to the surface of dental implants have shown a high capacity to accelerate osseointegration, compared with the traditional surfaces actually on the market, thanks to the formation of bone inside of the micropores, up to 200 microns [18–21].

In the light of what previously described, the choice of the right surgical approach, and of the best implants with the best characteristics, results to be the most important variable, in order to ensure a successful implant surgery and a proper prosthetic rehabilitation, also in those complexes cases, as illustrated.

## 5. Conclusion

The complex implant-prosthetic rehabilitations are a challenge for the surgeon and the prosthetist, creating critical issues both functional and aesthetic. However, these critical situations can be optimally managed, thanks to a proper presurgical planning.

With the planning of the case, surgery of implants can be carried out, respecting the surrounding biological structures.

Lastly, new dental implants with highly bioactive surfaces have been developed, providing an excellent and rapid bone integration.

Despite these new technologies as well as technologically advanced products, the surgeon should monitor the plants for a period of no less than six years.
